# Live Imaging Transverse Sections of Zebrafish Embryo Explants

**DOI:** 10.21769/BioProtoc.4928

**Published:** 2024-02-05

**Authors:** Eric Paulissen, Benjamin L. Martin

**Affiliations:** Department of Biochemistry and Cell Biology, Stony Brook University, Stony Brook, NY, USA

**Keywords:** Zebrafish explants, Mesoderm, Fluorescent microscopy, Deep tissues, Morphogenesis, Imaging

## Abstract

Vertebrate embryogenesis is a highly dynamic process involving coordinated cell and tissue movements that generate the final embryonic body plan. Many of these movements are difficult to image at high resolution because they occur deep within the embryo along the midline, causing light scattering and requiring longer working distances. Here, we present an explant-based method to image transverse cross sections of living zebrafish embryos. This method allows for the capture of all cell movements at high-resolution throughout the embryonic trunk, including hard-to-image deep tissues. This technique offers an alternative to expensive or computationally difficult microscopy methods.

Key features

• Generates intact zebrafish explants with minimal tissue disturbance.

• Allows for live imaging of deep tissues normally obscured by common confocal microscopy techniques.

• Immobilizes tissues for extended periods required for time-lapse imaging.

• Utilizes readily available reagents and tools, which can minimize the time and cost of the procedure.

**Graphical overview**


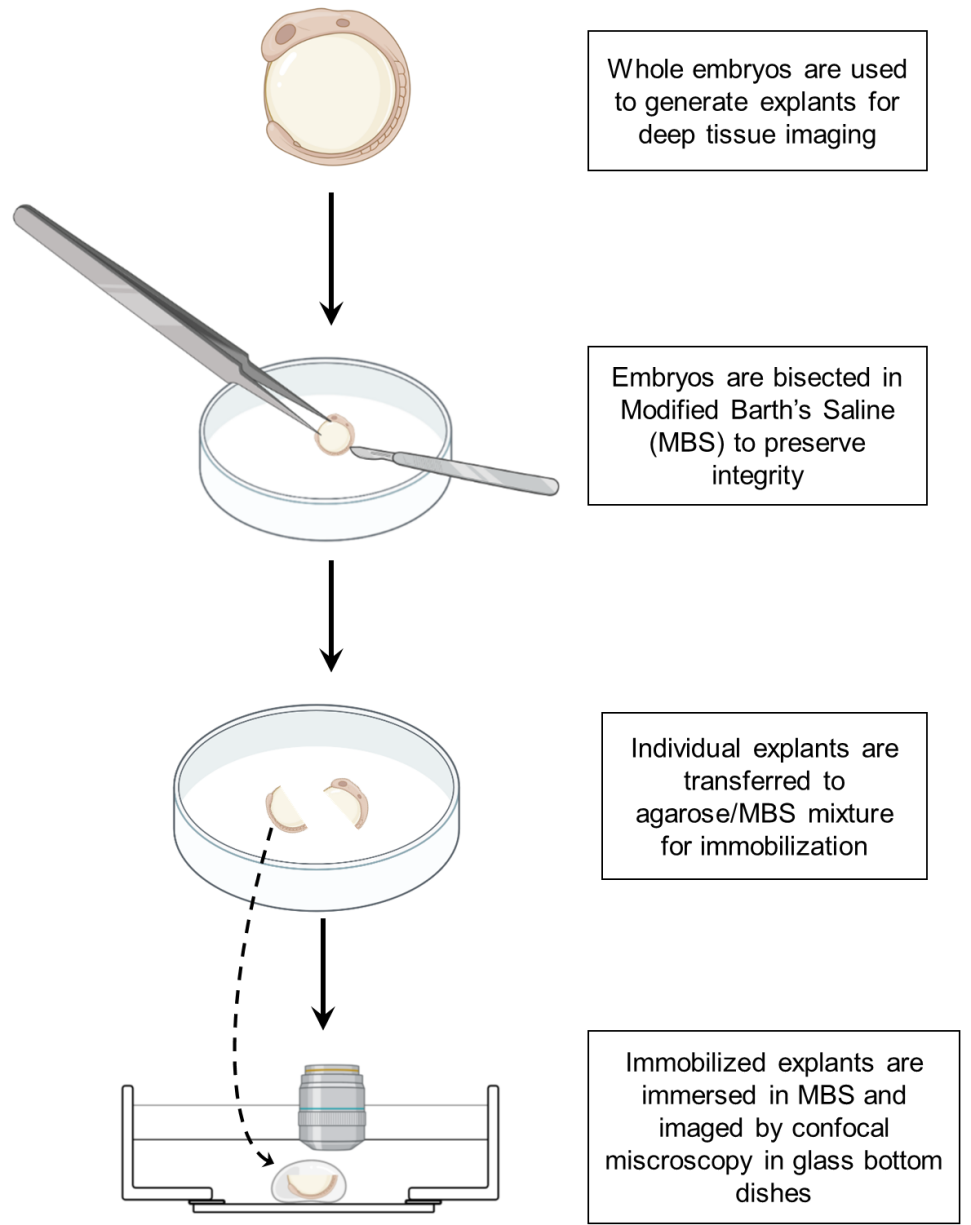


## Background

A central question in developmental biology is understanding how embryonic tissues organize themselves into the correct form. Embryos go through a series of cell/tissue interactions and broad dynamic movements to adopt their final shape. However, observing embryonic tissues in three-dimensional space can be challenging. Zebrafish are an excellent model system for visualizing living tissues. The relative transparency of zebrafish embryos compared to other model systems facilitates imaging of deeper tissues, and transgenic reporter lines allow for the visualization of specific cell or tissue types (Kimmel et al., 1995 ; Tonelli et al., 2017).

Despite the advantages of the zebrafish model, deep tissues can still be difficult to image at high resolution due to light scattering and the long working distance that is required (Jonkman and Brown, 2015). These issues can prevent detailed analysis of cell and tissue movements deep within a zebrafish embryo, such as the notochord or other tissues at the embryonic midline. Imaging of transverse sections can alleviate these problems, but prior methods require fixation and thus prohibit analyzing dynamic tissue movements in real time. We therefore developed a novel method of imaging living transverse sections of zebrafish using explants.

Zebrafish explants have been used frequently to understand various aspects of development (Langenberg et al., 2003; Picker et al., 2009; Simsek and Özbudak, 2021). Often, explants are used to separate tissues away from signaling sources or to conform tissues for specific analyses (Manning and Kimelman, 2015; Schauer et al., 2020). However, our methodology provides a novel way to orient intact embryonic trunk explants to image tissues of a transverse section. We first used this method to make time-lapse movies of the migration of angioblasts from bilateral regions of the embryo to the midline, where they differentiate into the dorsal aorta and cardinal vein (Paulissen et al., 2022). An advantage of this method is that it keeps most of all trunk tissues intact including the portions of the yolk to help stabilize the embryo. It allows for straightforward observation of cellular activity along the entire dorsal–ventral and medio–lateral axes, which would otherwise be difficult to capture. It also has an added benefit of using serum-less culture media that can be heated without worry of denaturation, which is relatively inexpensive compared to other methods that require serum-based media. Taken together, this new method provides an inexpensive, straightforward approach for visualizing deep-tissue movements at high-resolution during zebrafish embryogenesis.

## Materials and reagents

**Biological materials**

1. Wild-type embryos used in this study were from hybrid adults generated from an inbred strain of locally acquired pet store fish (which we call Brian) crossed to the TL line (to generate TLB)
2. The *tg(hsp70l:CAAX-mCherry-2A-NLS-KikGR)^sbu104^* transgenic strain was maintained on the TLB background (Goto et al., 2017). Our lab can provide this strain through resource sharing; however, many commercially available reporter lines can be used to label tissues of interest, including through the Zebrafish International Resource Center (ZIRC)

**Reagents**

1. NaCl (Millipore Sigma, catalog number: S9888)
2. KCl (Millipore Sigma, catalog number: P3911)
3. CaCl·2H_2_O (Millipore Sigma, catalog number: C7902)
4. KH_2_PO_4_ (Millipore Sigma, catalog number: P5655)
5. NaHPO_4_ (Millipore Sigma, catalog number: S5011)
6. MgSO_4_·7H_2_O (Millipore Sigma, catalog number: M1880)
7. Modified Barth's saline (1×) (MBS), liquid, without Ficoll^TM^ 400 (Millipore Sigma, catalog number: F-04-B)
8. Agarose, low-gelling temperature (Millipore Sigma, catalog number: A4018)

**Solutions**

1. Embryo growth medium (see Recipes)
2. 1.2% low-gelling agarose in MBS (see Recipes)

**Recipes**

1. Embryo growth medium
  **Table:** 

Reagent	Final concentration	Quantity
NaCl	15 mM	N/A
KCl CaCl·2H_2_O KH_2_PO_4_ NaHPO_4_ MgSO_4_·7H_2_O	0.5 mM 1.3 mM 0.15 mM 0.05 mM 1.0 mM	N/A N/A N/A N/A N/A
H_2_O	n/a	Desired volume (usually 50 mL per clutch of eggs laid)
2. 1.2% low-gelling agarose in MBS
  **Table:** 

Reagent	Final concentration		Quantity
MBS		N/A	100 mL
Low gelling agarose		1.2%	1.2 g
Total		N/A	100 mL

**Laboratory supplies**

1. Pyrex reusable Petri dishes, 100 mm (Fisher Scientific, catalog number: 08-747B)
2. Fisherbrand polystyrene Petri dishes, 100 mm × 15 mm (Fisher Scientific, catalog number: FB0875713)
3. Glass-bottom dish, 50 mm No. 1.5 coverslip 30 mm glass diameter uncoated (Mattek, catalog number: P50G-1.5-30-F)
4. Nunc^TM^ 15mL conical sterile polypropylene centrifuge tubes (Thermo Scientific, catalog number: 339650)
5. General purpose water bath set to 42 °C (Thermo Scientific, catalog number: TSGP20)
6. Microknives plastic handle, 22.5 degree cutting angle (Fine Science Tools, catalog number: 10316-14)
7. Dumont biological-grade forceps (Dumont, catalog number: 72700-D)
8. Bel-Art pipette pump 10 mL pipettor (Bel-Art, catalog number: F37898-0000)
9. Fisherbrand^TM^ disposable borosilicate glass Pasteur pipettes (Fisher Scientific, catalog number:13-678-20D)

## Equipment

1. Custom assembled spinning disk confocal microscope consisting of an automated Zeiss frame, a Yokogawa CSU-10 spinning disc, a Ludl stage controlled by a Ludl MAC6000, and an ASI filter turret mated to a Photometrics Prime 95B camera
2. Zeiss Plan-Apochromat 40× Dipping Microscope Objective (Zeiss, catalog number: 421462-9900-799)
3. Leica S9E stereoscope with a Leica KL300 LED light source (Leica)

## Software

1. The spinning disc microscope was controlled with Metamorph microscope control software and images were obtained with Metamorph (V7.10.2.240 Molecular Devices)

## Procedure

- **Preparing embryos to generate trunk explants**
  This process details the preparatory steps for generating trunk explants. This includes the steps up to the point at which the embryo is cut.
  1. Collect and store freshly laid embryos in polystyrene Petri dishes in embryo growth medium until roughly 2 h before the desired stage for sectioning.
  2. Pyrex reusable dishes should be clean and sterile before sectioning process begins. Brand new dishes are preferable.
  3. Prepare modified Barth’s saline (MBS) with low-gelling agarose. Melt 1.2% agarose powder into MBS in a laboratory microwave, avoiding boiling off the liquid as much as possible.
  4. Place melted MBS/agarose mixture in a 15 mL polypropylene conical tube in a 42 °C water bath to prevent solidification. Allow at least 30 min for the MBS/agarose mixture to equilibrate to 42 °C.
  5. Add approximately 25 mL of MBS to a clean Pyrex reusable dish.
  6. Transfer embryos to be sectioned from embryo growth media to the Pyrex dish containing MBS using a fire-polished glass Pasteur pipette. Low amounts of embryo growth media (< 100 μL) are tolerated in the MBS. If this cannot be achieved, MBS should be changed to fresh media.
  7. Using clean Dumont forceps, manually remove chorions from the embryos in the MBS media.
- **Sectioning and mounting trunk explants**
  This step is for the sectioning and mounting of embryo explants. The following steps require both speed and precision to prevent excess damage to the explant. Individual explants should be mounted soon after sectioning before continuing to the next explant. Our protocol maintains the yolk to allow for more accurate morphology and to prevent distention during mounting. All subsequent steps should be performed under a dissection stereomicroscope. We utilize the Leica S9E stereomicroscope, but most commercial stereomicroscopes are sufficient.
  1. Using two Dumont forceps, carefully open a section of the yolk away from the sectioning area (see Figure 1A). This allows yolk to vent away from the sectioning area to prevent obscuring of the trunk region during imaging as well as preventing endoderm damage (Figure 1A and 1B).
    **Figure 1.**
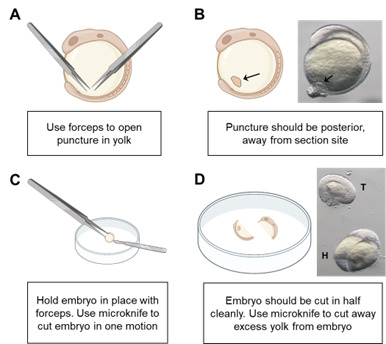
Schematic of embryo section. A. Opening a puncture in the yolk of the embryo allows venting of yolk after sectioning. This reduces the need to remove yolk manually, which can damage the endoderm. Use forceps to puncture yolk. B. Image of puncture in an 8-somite-stage embryo (black arrows). C. Schematic showing the position of forceps and microknife during sectioning. D. Image of sectioned explant. Tail of the explant (T) and head of explant (H) are visible.
  2. Immediately afterward, using the forceps to immobilize the embryo, section the area cleanly using a microknife (Figure 1C and 1D).
  3. Remove the agarose/MBS mixture from the 42 °C water bath. Add a droplet of agarose/MBS (roughly 100 μL) to a Mattek glass-bottom dish. Quickly and carefully transfer the explant to the droplet using a fire-polished Pasteur pipette while avoiding adding too much MBS during the transfer (Figure 2A and 2B).
    **Figure 2.**
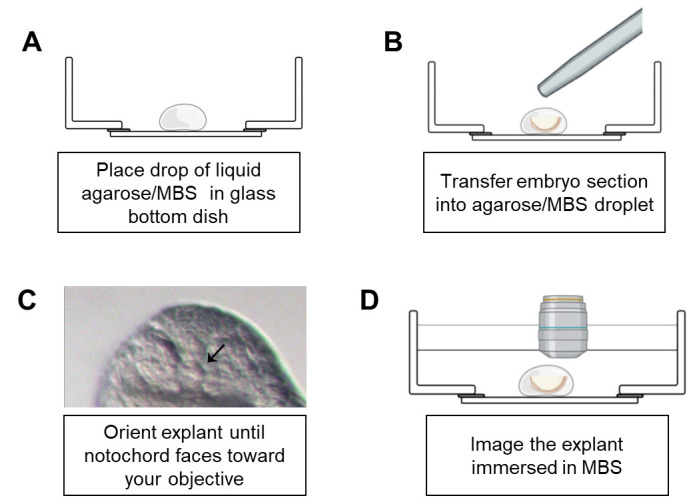
Schematic of embryo mounting in glass-bottom dish. A. Place a liquid drop of agarose/MBS on a glass-bottom dish. B. Immediately transfer the desired explant to the drop using a fire-polished pipette. C. Using forceps, gently orient the embryo until you adopt the proper position. A good marker of correct orientation is seeing a visible round notochord through the dissecting scope, as shown in the image (black arrow). D. Add MBS to cover the surface of the solidified agarose/MBS droplet. Using dip lenses may be necessary to image explant.
  4. Prior to the agarose solidifying, carefully orient the explant using your Dumont forceps such that the transverse section is visible through the stereomicroscope (Figure 2C). Hold the embryo in place until the agarose cools.
  5. Carefully remove your forceps from the droplet. Add additional liquid agarose/MBS to the periphery of the droplet if further stabilization of the explant is required; this can range from 100 to 500 μL as desired.
  6. Let the agarose cool to solidification. Add additional MBS over explant until it is completely submerged; this is usually 2 mL but can depend on agarose volume added in previous step. The more agarose added, the less volume of MBS is required.
- **Imaging trunk explants**
  Imaging explants requires an inverted microscope or an upright microscope using water-immersion dipping objectives. Each methodology has strengths and weaknesses. With an inverted microscope, there is no need for specialized objectives when using the glass-bottom dishes. However, orienting the explant can be more challenging when using an inverted microscope, and the explant must also be pressed close to the coverslip. By contrast, an upright microscope works best in this context when using water immersion lenses that allow submersion in culture media. In this case, it is easier to correctly orient the explant (Figure 2D). Take care to not add excessive MBS to the glass-bottom dish to avoid spilling into the base of the microscope when the lens enters the media. Explants mounted by this method can last 6–8 h during timelapse.
  Using this method can bring to light cellular movements that were previously difficult to document. This method was instrumental in our ability to visualize the migration of angioblasts from bilateral positions to the embryonic midline (Paulissen et al., 2022). Here, we provide another example of deep tissue imaging using this explant strategy. For our example, we image the developing neural tube as it transitions from the neural keel to the neural rod (Cearns et al., 2016). The developing neural tube is a large structure that can be difficult to image at high resolution without sectioning. Using a transgenic embryo that labels the cell membranes, *tg(hsp70l:CAAX-mCherry-2A-NLS-KikGR)^sbu104^*, we were able to observe cells as they transitioned from an unpolarized to polarized state (Figure 3A-3C, Supplemental Video 1).
  **Figure 3.**
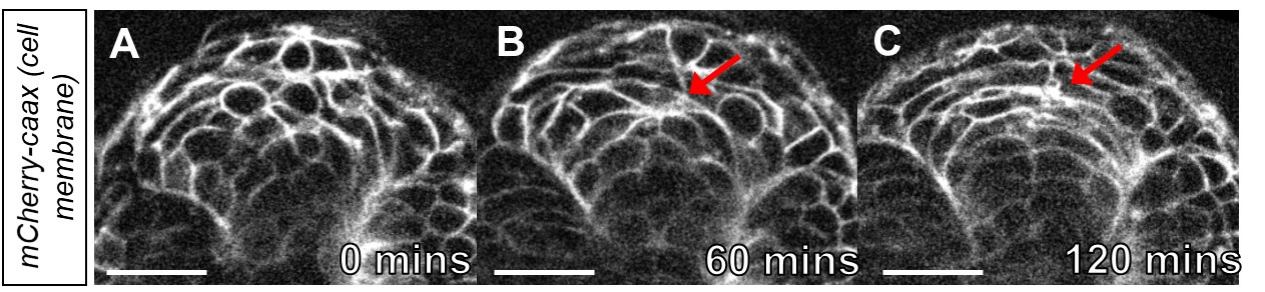
Time-lapse imaging of cell polarization in the neural keel. A. Image of the neural keel starting at the 10-somite stage. B. Image of the neural keel after 60 min. Note the polarization of cells at the midline (red arrow). C. Image of neural keel after 120 min observation during the transition to the neural rod. Note the cells have polarized at the midline (red arrow ). Scale bars = 50 μm.
**Considerations and limitations**
  This technique allows for long-term imaging of embryo explants starting from early somitogenesis stages. Explants from early to mid-somitogenesis stages can be imaged over an 8 h period before they begin to lose their fidelity. Explants from older embryos (24 hpf or older) can survive longer (up to 18 h) after being generated. The initial survivability of explants can vary depending on sectioning precision and the amount of agitation that occurs when transferring explants. If transferred carefully with minimal agitation, you can expect survivability of >50%. Explants, particularly at earlier stages, will still undergo broad morphogenetic movement including axial extension, which can cause the region of interest to move out of focus during time-lapse imaging. Removing unnecessary tissues like the tailbud can prevent this movement, which we demonstrated in a previous study (Paulissen et al., 2022). Anterior half explants containing the head, rather than the posterior half containing the tailbud, have more limited anterior–posterior axis extension and are preferable for imaging. We also note that these spatial issues can occur even with whole, intact embryos, and are not unique to this explanation method (Hirsinger and Steventon, 2017). Sectioning and explant generation can be performed at variable positions along the anterior-posterior axis as well as multiple positions. However, we recommend limiting this to two sectioning positions within one embryo as smaller explants are more difficult to transfer and orient.

## Validation of protocol

The protocol presented here was performed previously in the reference listed below. A total of 12 explants were imaged (4 explants on three separate occasions).

- Paulissen, E., Palmisano, N. J., Waxman, J. S. and Martin, B. L. (2022). Somite morphogenesis is required for axial blood vessel formation during zebrafish embryogenesis. *eLife* 11: e74821. (Figure 7, panel O, Video 8, Video 9).

## Supplementary information

The following supporting information can be downloaded here:
